# Correction: Neukart et al. Extending the QMM Framework to the Strong and Weak Interactions. *Entropy* 2025, *27*, 153

**DOI:** 10.3390/e28070791

**Published:** 2026-07-13

**Authors:** Florian Neukart, Eike Marx, Valerii Vinokur

**Affiliations:** 1 Leiden Institute of Advanced Computer Science, Leiden University, Gorlaeus Gebouw-BE-Vleugel, Einsteinweg 55, 2333 Leiden, The Netherlands; 2 Terra Quantum AG, Kornhausstrasse 25, 9000 St. Gallen, Switzerland; eike@terraquantum.swiss (E.M.);

## Reference Correction

In the original publication [1], there were errors in References [21–24]. In each affected entry, the journal, volume, article/page number, and/or DOI corresponded to an existing publication, but the author names and article titles in the reference list were incorrect. The affected entries should be corrected as follows:

○ Reference 21

Original entry:

Doe, J.; Roe, A. Hollow Quantum Shells in Black Hole Formation. *Int. J. Mod. Phys. D* **2014**, *23*, 1441002.

Corrected entry:

Vaz, C. Black Holes as Gravitational Atoms. *Int. J. Mod. Phys. D* **2014**, *23*, 1441002. https://doi.org/10.1142/S0218271814410028.

○ Reference 22

Original entry:

Roe, A.; Smith, B.; Zhang, C. Strong Quantum Fluctuations in Hollow Black Holes. *Commun. Theor. Phys*. **2023**, *75*, 095405.

Corrected entry:

Corda, C. Schrödinger and Klein-Gordon Theories of Black Holes from the Quantization of the Oppenheimer and Snyder Gravitational Collapse. *Commun. Theor. Phys.* **2023**, *75*, 095405. https://doi.org/10.1088/1572-9494/ace4b2.

○ Reference 23

Original entry:

Doe, J.; Roe, A.; Smith, B. Quantum Shell Description of Schwarzschild Horizons. *Int. J. Mod. Phys. D* **2022**, *31*, 2230006.

Corrected entry:

Ha, Y.K. The Irreducible Mass of Christodoulou-Ruffini-Hawking Mass Formula. *Int. J. Mod. Phys. D* **2022**, *31*, 2230006. https://doi.org/10.1142/S0218271822300063.

○ Reference 24

Original entry:

Smith, B.; Zhang, C.; Doe, J. Black Holes as Condensed Quantum Shells. *Phys. Lett. B* **2024**, *856*, 138948.

Corrected entry:

Corda, C.; Cafaro, C. Universality of the thermodynamics of a quantum-mechanically radiating black hole departing from thermality. *Phys. Lett. B* **2024**, *856*, 138948. https://doi.org/10.1016/j.physletb.2024.138948.

The order of References [21–24] remains unchanged. These corrections are necessary to ensure that the published reference list accurately reflects the underlying primary publication records. The correction concerns bibliographic entries only. The authors state that the scientific conclusions are unaffected. This correction was approved by the Academic Editor. The original publication has also been updated.
